# Adverse events associated with systemic cutaneous T-cell lymphoma therapies: a narrative review

**DOI:** 10.3389/fmed.2026.1912209

**Published:** 2026-09-07

**Authors:** Krithika Nayudu, Beatrix B. Thompson, Cecilia Larocca

**Affiliations:** 1 Medical College of Georgia, Augusta, GA, United States; 2 Center for Cutaneous Oncology, Dana Farber Cancer Institute, Boston, MA, United States; 3 Harvard Medical School, Boston, MA, United States

**Keywords:** adverse events, cutaneous lymphoma, mycosis fungoides, sezary syndrome (SS), systemic therapies

## Abstract

**Background:**

Mycosis fungoides (MF) and Sézary syndrome (SS) are the most prevalent subtypes of cutaneous T-cell lymphoma (CTCL). Systemic therapies span multiple drug classes, and as most are administered with palliative intent over prolonged periods, the adverse event (AE) profile of each agent is as clinically important as its efficacy. No comprehensive narrative review has synthesized AE data across all National Comprehensive Cancer Network (NCCN)-recommended systemic therapies for MF/SS.

**Objectives:**

To summarize and compare the AE profiles of all 14 NCCN guideline-recommended systemic therapies for MF and SS, with emphasis on cutaneous toxicities and their distinction from active disease.

**Methods:**

Systemic therapies were identified from Version 2.2026 NCCN Guidelines for Cutaneous Lymphomas. Safety data were abstracted from available Phase II and III trials supporting guideline inclusion, with attention to AE frequency, severity, dose-limiting toxicities, and treatment discontinuation rates. PubMed and Scopus were searched for case reports and series capturing rare or delayed toxicities not represented in prospective trials. Data were narratively synthesized given heterogeneity across study designs and reporting practices.

**Results:**

Fourteen NCCN-recommended systemic agents were reviewed, spanning antibody-drug conjugates, monoclonal antibodies, histone deacetylase (HDAC) inhibitors, retinoids, antifolates, immunotoxins, cytotoxic chemotherapies, interferons, and immune checkpoint inhibitors. Toxicity profiles varied substantially by drug class. Peripheral neuropathy was the defining AE of brentuximab vedotin. Mogamulizumab was distinguished by mogamulizumab-associated rash, a treatment-emergent immune reaction that closely mimics CTCL progression. Bexarotene required proactive management of hypertriglyceridemia and central hypothyroidism. HDAC inhibitors carried gastrointestinal, hematologic, and cardiac risks, while cytotoxic agents posed variable risks of myelosuppression, mucositis, and hepatotoxicity. Immunomodulatory agents introduced risks of opportunistic infection and paradoxical disease flares.

**Conclusions:**

Systemic therapies for MF/SS carry distinct AE profiles that should inform treatment selection, patient counseling, and monitoring. A recurring challenge is distinguishing cutaneous drug toxicity from CTCL progression, underscoring the importance of therapy-specific toxicity awareness. Standardized AE reporting and patient-centered outcome measures are needed to optimize long-term care in this population.

## Introduction

Mycosis fungoides (MF) and Sézary syndrome (SS) are the most prevalent subtypes of cutaneous T-cell lymphoma (CTCL), accounting for approximately 50% and 3%–5% of all cutaneous lymphoma cases, respectively (1). MF typically follows an indolent course, presenting with patches, plaques, and tumors that may remain confined to the skin for decades, while SS is the aggressive, leukemic variant characterized by erythroderma and circulating malignant T-cells with a significantly worse prognosis (2).

Systemic treatment of MF and SS encompasses a wide and heterogeneous arsenal of agents spanning multiple drug classes, including anti-CD30 antibody-drug conjugates, anti-CCR4 monoclonal antibodies, antifolate agents, histone deacetylase (HDAC) inhibitors, and immunotoxins (3). Despite the breadth of available therapies, definitive cure remains elusive for most patients with MF/SS beyond allogeneic stem cell transplantation, and systemic therapy is typically administered with palliative intent over prolonged periods. In this context, the adverse event (AE) profile of each agent is as clinically important as its efficacy, directly influencing quality of life and treatment adherence. Pruritus, a near-universal and frequently debilitating symptom of MF/SS, adds additional complexity to AE assessment, as it may reflect active disease, drug effects, or both (4).

No comprehensive narrative review has synthesized the AE profiles of all systemic therapies for MF/SS recommended by the National Comprehensive Cancer Network (NCCN). This narrative review aims to summarize evidence on AE from Phase II and III trials, case series, and case reports on systemic CTCL therapies, serving as a reference for trainees and faculty involved in the complex management of MF/SS patients.

## Methods

Systemic therapies for CTCL were identified from Version 2.2026 of the NCCN Guidelines for Cutaneous Lymphomas (3). Therapies were limited to those included as current NCCN-recommended systemic agents for MF/SS. Safety data were abstracted from the available phase II and phase III studies which supported guideline inclusion for each therapy, with attention to AE frequency, severity, dose-limiting toxicities, rates of treatment discontinuation, and distinctive toxicities. The Common Terminology Criteria for Adverse Events (CTCAE) was the standard for grading the severity of AEs: Grade 1 (mild) refers to events with asymptomatic or mild symptoms which necessitated clinical or diagnostic observations only or for which interventions were not indicated; Grade 2 (moderate) refers to events for which minimal, local, or noninvasive intervention was indicated or which limited age appropriate instrumental activities of daily living (ADL); Grade 3 (severe or medically significant but not immediately life threatening) refers to events for which hospitalization or prolongation of hospitalization was indicated or which were disabling or limited self-care ADL; Grade 4 (life-threatening consequences) refers to events for which urgent intervention was indicated; and Grade 5 refers to death related to the AE (5).

To capture rare or delayed AE or toxicities not represented in prospective trials, PubMed and Scopus were searched for peer-reviewed case reports and series describing AEs associated with each included therapy, up until 6/10/2026. Search terms combined each systemic therapy term with “cutaneous T-cell lymphoma,” “mycosis fungoides,” “Sézary syndrome,” “adverse event,” “toxicity,” “rash,” “eruption,” and related toxicity-specific terms when applicable. Relevant English-language articles were reviewed, from which adverse events were extracted.

Data were narratively synthesized due to the inherent variability in study design, CTCL subtype and stage, prior treatment exposure, dosing regimens, adverse event grading systems, and reporting practices across studies. Table 1 summarizes the overall response rates (ORR), most common adverse events, and high-grade toxicities reported in the reviewed literature.

**Table 1:** Adverse events (AEs) from pivotal trials of established MF/SS systemic therapies.

Drug (Key trial)	*N* ^a^	ORR (%)^b^	Grade ≥3 AEs	Cutaneous AEs	Common non-cutaneous AEs (> 10%)
DD-cxcl (6)	69	36.2	43.5% overall (ALT elevation 8.7%, infusion reaction 5.8%, pruritus 5.8%, capillary leak syndrome 5.8%, AST elevation 4.3%)	Pruritus (18.8%)	Nausea (43.5%), fatigue (31.9%), ALT elevation (27.5%), chills (27.5%), peripheral edema (27.5%), AST elevation (26.1%), infusion-related reaction (24.6%), headache (23.2%), diarrhea (18.8%), capillary leak syndrome (17.4%), pyrexia (15.9%), hypoalbuminemia (14.5%), decreased appetite (13.0%), dizziness (13.0%), blurred vision (13.0%), vomiting (13.0%), weight increased (13.0%), arthralgia (11.6%), constipation (11.6%), myalgia (11.6%)
Gemcitabine (7)	33	68	39.4% overall (leukopenia 18.2%, cutaneous hyperpigmentation 15.2%, thrombocytopenia 12.1%, anemia 9.1%)	Cutaneous hyperpigmentation (18.2%), radiation recall ulceration (3.0%)	Myalgia/fatigue (21.2%), fever (24.2%), nausea (15.1%), chills (12.1%), increased ALT/AST (12.1%)
Gemcitabine (8)	32	75	34.4% overall (leukopenia 16%, thrombocytopenia 12%, hepatic 6%, anemia 3%)	None reported	Anemia (40.5%), thrombocytopenia (56%), leukopenia (53.5%), hepatotoxicity (40.6%)
Pegylated liposomal doxorubicin (9)^d^	49	40.8	Other dermatologic toxicity (6.1%), infection (4%), constitutional symptoms (4%), GI toxicity (4%), hand-foot reaction (2%), cardiac (2%), allergy (2%), PE (2%), cardiac ischemia (2%)	Hand-foot reaction (2% grade 3), other dermatologic toxicity (6% grade 3)	None reported
Interferon alpha (10)	22	64	CTCAE grading not used; Elevated AST >2 × normal (9%)	Hair loss (23%)	Fatigue (100%), fevers/chills (86%), myalgias (55%), anorexia (55%), weight loss (55%), nausea (45%), depression (36%), cough (32%), headache (27%), bad/bitter taste (23%), abdominal pain (18%), dry mouth (18%), diarrhea (14%)
Interferon gamma (11)	16	31	CTCAE grading not used; “Clinical toxic effects”: Neutropenia ≤2,500/mm^3^ (38%), nephrotic syndrome (6%), allergic cutaneous reaction (6%)	Cutaneous allergic reaction (6%)	Elevated AST (100%), elevated lactate dehydrogenase (100%), fever (69%), weight loss (69%), marked weight loss >%5 (38%), neutropenia (38%)
Alemtuzumab (12)	22	55	Infectious adverse events (45%); Hematologic adverse events (23%)	Rash/urticaria (14%)	Anemia (100%; no grade 4), neutropenia (100%, 18% grade 4), thrombocytopenia (100%, 5% grade 4), infection (45%), including cytomegalovirus reactivation (18%)
Pembrolizumab (13)	24	38	Cutaneous adverse events (16.7%), arthritis, pneumonitis, colitis/duodenitis, elevated AST, elevated ALT, corneal ulcer, Pneumocystis pneumonia (G4), anemia, hypotension, hypertension, pulmonary edema, periorbital edema (4.2% for all)	Rash/skin flare (25% grade 1–2, 16.7% grade 3); transient erythroderma/pruritus flare in 53% of SS patients	Anemia (12.5%), arthritis/arthralgia (12.5%)
Temozolomide (14)	26	27	Skin reaction (6.9%), infection (6.9%), thrombocytopenia, neutropenia, nausea/vomiting (3.4% for all)	Rash or erythema (53.8%), pain/pruritus (76.9%), alopecia (13.8%)	Fatigue (57.7%), fever/chills (38.5%), thrombocytopenia (38.5%), infections (34.6%), neutropenia (15.4%)
Brentuximab Vedotin (15)	66	67	41% overall (peripheral neuropathy 5%, fatigue 5%, diarrhea 3%, skin infection 3%, nausea 2%, vomiting 2%, pruritus 2%, asthenia 2%, maculopapular rash 2%, generalized pruritus 2%)	Alopecia (15%), pruritus (17%), maculopapular rash (11%), generalized pruritus (11%), skin infection (3%)	Peripheral neuropathy (45%), nausea (36%), diarrhea (29%), fatigue (29%), vomiting (17%), pruritus (17%), pyrexia (17%), alopecia (15%), decreased appetite (15%), arthralgia (12%), myalgia (12%), asthenia (11%), dyspnea (11%), maculopapular rash (11%), peripheral edema (11%), generalized pruritus (11%)
Mogamulizumab (16)	184	28	41% overall (hypertension 4%, drug eruption 4%, pneumonia 4%, cellulitis 3%, infusion-related reaction 2%, fatigue 2%, constipation 1%, diarrhea 1%, nausea 1%, pyrexia 1%, sepsis 1%, aspartate aminotransferase increased 1%, weight decreased 1%, decreased appetite 1%)	Drug eruption (23.9%), alopecia (7.1%), skin pain (3.3%), rash (3.3%), skin fissures (3.3%), skin ulcers (2.7%), dermatitis exfoliativa (1.6%), photosensitivity (1.1%), generalized rash (1.1%)	Infusion-related reaction (33.1%), drug eruption (23.9%), diarrhea (23.3%), fatigue (23.3%), pyrexia (16.8%), nausea (15.2%), peripheral edema (14.7%), headache (12.5%), constipation (11.4%), thrombocytopenia (11.4%), upper respiratory tract infection (10.3%), anemia (10.3%)
Pralatrexate (17)^c^	34	61	Neutropenia (35%), hypertriglyceridemia (29%), stomatitis (21%), peripheral edema (6%), skin infection (6%), diarrhea (6%), dyspnea (6%), dizziness (3%), anemia (3%)	Skin infection (26%)	Stomatitis (65%), fatigue (44%), nausea (32%), neutropenia (32%), anemia (24%)
Romidepsin (18)	96	34	Asthenic conditions (6%), nausea (2%), anemia (2%), tumor lysis syndrome (2%), vomiting, diarrhea, oral candidiasis, oropharyngeal candidiasis, anemia atrioventricular block, cardiac failure, cardiopulmonary failure, hyperglycemia, hypoalbuminemia, leukocytosis, pleural effusion, pyrexia, sepsis, dermatitis medicamentosa, ventricular tachycardia, troponin 1 increase, cardiac tamponade (1% for all)	Dermatitis medicamentosa (1%)	Nausea (56%), asthenic conditions (44%), vomiting (26%), anorexia (20%), diarrhea (14%), headache (14%), ageusia (13%), thrombocytopenia (11%), dysgeusia (11%), anemia (10%)
Vorinostat (19)	74	29.7	28% overall (fatigue 5.4%, thrombocytopenia 5.4%, nausea 4.1%, anorexia 2.7%, muscle spasms 2.7%, weight decrease 1.4%, blood creatinine increase 1.4%, anemia 1.4%, chills 1.4%)	Alopecia (17.6%)	Diarrhea (49%), fatigue (46%), nausea (43%), anorexia (26%), dysgeusia (24%), thrombocytopenia (22%), weight decrease (20%), alopecia (18%), muscle spasms (16%), blood creatinine increase (15%), anemia (12%), chills (12%), vomiting (12%), constipation (11%), dry mouth (11%)
Bexarotene (5, 20)^e^	56	45	CTCAE grading not used; “Severe” events: hyperlipemia 10.7%, pruritus 7.1%, rash 3.6%, skin disorder 3.6%, infection 1.8%, peripheral edema 1.8%, hypothyroidism 1.8%, dermatitis exfoliation 8.9%)	Dermatitis exfoliation (8.9%), pruritus (24.9%), rash (17.8%), skin disorder (16.1%)	Hyperlipemia (82%), hypercholesteremia (30%), hypothyroidism (29%), headache (20%), asthenia (16%), pruritus (13%), leukopenia (11%), skin disorder (11%), and rash (11%)

a *N* reflects safety population for all trials.

b Efficacy-evaluable populations used for ORR calculation: Brentuximab-vedotin (15) *n* = 64; Mogamulizumab (16) *n* = 186; Pralatrexate (17) *n* = 34; Temozolomide (Querfeld reported an ORR in 7/26 evaluable patients; Tani reported an ORR in 3/9 patients, bringing the pooled ORR to 28.6%).

c Only AEs explicitly attributed to pralatrexate were included.

d Only grade ≥3 clinical adverse events and grade ≥2 hematologic/biochemical events were captured per protocol; all-grade cutaneous AE percentages are not available.

e AE data for bexarotene reported only from group assigned to initial dose of 300 mg/m^2^/d.

## Results

Adverse events from fourteen NCCN-guideline recommended therapies were included and range across conventional cytotoxic chemotherapies, targeted therapies, immunotherapies, and antibody-drug conjugates.

### Brentuximab vedotin

Brentuximab vedotin (BV) is an antibody-drug conjugate comprised of an anti-CD30 antibody conjugated to monomethyl auristatin E (MMAE), a cytotoxic microtubule disruptor approved for the treatment of relapsed and refractory Hodgkin lymphoma and systemic anaplastic large-cell lymphoma (21, 22). Two pivotal clinical trials have established its AE profile in CTCL. An investigator-initiated phase II study by Kim et al. investigated BV in patients with MF and SS across a range of CD30 expression levels (23). Peripheral neuropathy was the most commonly reported drug-related AE, occurring in 66% of patients. Twelve of 21 affected patients developed grade ≥2 neuropathy. Six patients (19%) discontinued BV early due to toxicity, with peripheral neuropathy being the primary driver of dose modification and early termination. The most common cutaneous toxicity reported was alopecia in 7/32 (22%) patients. Skin eruption was the only grade ≥3 cutaneous AE reported. Overall, the AE profile in CTCL was described as manageable (23).

Subsequently, the ALCANZA trial was the first randomized, phase III study of a novel systemic agent versus standard therapy in CTCL, enrolling 128 patients with relapsed or refractory CD30-positive MF or primary cutaneous anaplastic large-cell lymphoma (pcALCL) who were randomized to BV (1.8 mg/kg every 3 weeks) or physician's choice (15). At a median follow-up of 22.9 months, the safety profile of BV was consistent with that of the phase II trial. Peripheral neuropathy was the most frequent and clinically significant AE, occurring in 67% (44/66) of patients in the BV arm, compared to only 6% (4/62) in the physician's choice arm (15). Among patients who developed neuropathy, 21 patients experienced grade 2 AEs and 6 experienced grade 3 AEs; no grade 4 peripheral neuropathy was reported. Grade ≥3 AEs occurred in 41% of BV-treated patients vs. 47% in the physician's choice arm, indicating that, despite higher overall AE frequency with BV, severe toxicity was not disproportionately increased. Other commonly reported AEs to BV of any grade in ALCANZA were nausea (36%), fatigue (29%), diarrhea (29%), vomiting (17%), alopecia (15%), pruritus (17%), and maculopapular rash (11%) (15). Hematologic toxicities were uncommon. One noteworthy clinical finding from ALCANZA was the 2% incidence of hypertriglyceridemia of any grade with BV treatment.

Cutaneous AEs were generally mild and manageable in the ALCANZA trial. Alopecia was reported in 10/66 (15%) of patients in the BV arm compared to 4% in the physician's choice arm; there was no grade ≥3 alopecia reported in any arm (15). Among patients treated with BV, pruritus of any grade was noted in 17%, including 1 patient who had grade 3 pruritus. Other infrequent cutaneous AEs reported included a maculopapular rash (11%), generalized pruritus (11%), and skin infection (3%). The two patients with skin infection were categorized as grade 3 (15).

Four case reports describe distinct cutaneous toxicities to BV in CTCL. Hand-foot syndrome with severe palmar-plantar desquamation developed after 3 cycles in a patient with MF, and splinter nail hemorrhages with painful nail bed disruption occurred after 15 cycles in a patient with SS, both cases representing late-onset cutaneous manifestations of cumulative MMAE-mediated toxicity (24, 25). Tumor lysis syndrome presenting with lymphadenopathy and yellowish skin discoloration was reported the day after treatment initiation in a patient with MF (26). Alopecia has additionally been reported, consistent with ALCANZA's findings and with MMAE's known toxic effects on hair follicles (27). Non-cutaneous toxicities reported include pancreatitis, pneumonitis with organizing pneumonia, progressive multifocal leukoencephalopathy, and severe insulin resistance (28–32).

### Mogamulizumab

Mogamulizumab is a monoclonal antibody targeting CCR4, a marker often overexpressed on malignant T-cells (33). The MAVORIC trial compared mogamulizumab (1 mg/kg IV) to vorinostat in 372 patients with relapsed or refractory MF or SS. Cutaneous AEs emerged as the most clinically distinctive and challenging toxicities to identify and treat (16).

Drug eruption, now formally termed mogamulizumab-associated rash (MAR), was the single most frequently reported AEs in the mogamulizumab arm of MAVORIC, occurring in 20% of patients (16). MAR reflects a treatment-emergent immune-mediated skin reaction driven by T-reg depletion and aberrant T-cell activation directed against self-antigens in the skin (34). The unique diagnostic challenge of MAR is its close clinical and histopathologic resemblance to CTCL skin involvement. This distinction carries profound treatment implications, as misclassifying MAR as CTCL progression might lead to unnecessary therapy escalation, while failing to recognize CTCL progression as such may delay appropriate intervention.

Among other commonly reported AEs in the MAVORIC trial, infusion-related reactions occurred in 32% of patients; diarrhea (>23% of patients, generally grade 1–2), fatigue (22%), and pyrexia (16%) were notable (16). Grade ≥3 AEs occurred in 41% of both arms. Serious AEs were more frequent with mogamulizumab (38%) than vorinostat (25%), with pyrexia (4%) and cellulitis (3%) the most common serious events in the mogamulizumab group (16).

The case report literature is dominated by cutaneous toxicities and more rarely, delayed-onset mogamulizumab-related myositis or myocarditis (MAM/Mc). MAR has been reported across multiple morphologic variants including classic, acneiform, lichenoid/mucocutaneous, interface dermatitis, and alopecia areata-like patterns; MAR recurrence upon rechallenge has also been described (35–43). Additional cutaneous irAEs include conventional alopecia areata, scarring alopecia, vitiligo, generalized eruptive lentiginosis, and crusted scabies secondary to mogamulizumab-mediated immunosuppression (44–48). A single-center study of 39 patients identified immune-related AEs in 16 (41%) CTCL patients treated with mogamulizumab; thirteen (33.3%) patients developed MAR and 5 (12.9%) developed MAM/Mc (42). MAM/Mc were predominantly delayed toxicities, with a median onset time of 168 days following initiation of mogamulizumab, and in three cases, developed well after mogamulizumab discontinuation (43). IVIG alongside systemic corticosteroids was demonstrated to effectively and safely treat these potentially fatal toxicities (43). Other, non-cutaneous toxicities detailed in case reports and series include polymyalgia rheumatica, seropositive myasthenia gravis, inflammatory bowel disease, autoimmune hemolytic anemia, lymphadenopathy, and BK polyomavirus-associated progressive multifocal leukoencephalopathy (PML) (42, 49–52).

### Pralatrexate

Pralatrexate is an oral antifolate with high affinity for the reduced folate carrier-1 (RFC-1), enabling preferential uptake and polyglutamylation in tumor cells and greater intracellular retention compared with methotrexate (53). Its AE profile in CTCL was characterized in the phase I/II open-label, dose-finding study by Duvic et al., which examined pralatrexate in combination with oral bexarotene in 34 patients with relapsed/refractory CTCL (17). Pralatrexate was administered at 15 mg/m^2^ IV weekly for 3 of every 4 weeks alongside daily bexarotene at 150 mg/m^2^, with mandatory folic acid and intramuscular B12 supplementation beginning at least 10 days before the first dose (17).

Stomatitis was the most common and dose-limiting AE attributed to pralatrexate, occurring at any grade in 65% of patients in the Duvic et al. phase I/II study (17). Mucositis is posited to arise from pralatrexate's antiproliferative effects on rapidly dividing mucosal epithelium via the same RFC-1-mediated uptake that confers antitumor activity. Clinically, oral mucositis ranges from painless erythema and aphthous-like ulceration to confluent mucosal breakdown compromising oral intake.

The 15 mg/m^2^ CTCL-optimized dose showed relatively higher mucositis tolerability compared with the 30 mg/m^2^ PTCL dose, where mucositis occurred in approximately 71% of patients and dose omissions were required in 68% in the PROPEL trial (53). As such, oral mucositis severity should be assessed before each pralatrexate dose, and it is recommended to delay treatment for grade 2 or higher events until recovery to grade 1 or below.

Additional AEs attributed to pralatrexate by investigators in the phase I/II trial included fatigue (44%), nausea (32%), neutropenia (32%), and anemia (24%). Grade ≥3 hematologic toxicities were uncommon at the 15 mg/m^2^ dose, with grade 3 neutropenia in 32% and grade 4 leukopenia in 3% (17). Mandatory folate and B12 supplementation is required to mitigate both mucocutaneous and hematologic toxicities (17).

### Methotrexate

Methotrexate (MTX) is a classic oral antifolate used in patients with CTCL at low weekly oral or intramuscular doses (5–75 mg/week). The most rigorous prospective evidence supporting MTX's use in CTCL comes from a head-to-head randomized trial comparing MTX to interferon-alpha as first-line systemic therapy in 43 patients with advanced MF and Sézary syndrome (54). While not a classic single-arm phase II trial, this study provides the most controlled available prospective data on MTX tolerability in CTCL, reporting AEs in 81% of MTX-treated patients, which was significantly higher than in the IFN-α arm (*p* = 0.02) (54). Abnormal liver function tests, hepatic toxicity, and cirrhosis were the most frequent AEs, occurring more often than others such as myalgia, arthralgia, and osteoporosis (*p* = 0.01) (54). The majority of the MTX evidence base in CTCL consists of retrospective observational series, which characterize a toxicity profile including mucocutaneous ulceration, hepatotoxicity, myelosuppression, pulmonary toxicity, and nephrotoxicity (55, 56). Several of these toxicities, particularly mucocutaneous ulceration and cutaneous necrosis, carry heightened significance in CTCL patients given the complexity of distinguishing drug-related skin toxicity from underlying disease.

Case reports describe a range of serious MTX-associated AEs in CTCL. Secondary lymphoproliferative disorders, including MTX-related Epstein-Barr virus (EBV)-positive diffuse large B-cell lymphoma and B-cell lymphoproliferative disease, highlight the rare, but possible malignancy risk related to chronic MTX-mediated immunosuppression (57, 58). Cutaneous cryptococcosis has been reported, which presented as cutaneous nodules in a CTCL patient on low-dose MTX (59). MTX-induced cutaneous ulceration superimposed on erythrodermic skin, requiring differentiation from disease progression, has additionally been documented in a case series of erythrodermic MF patients (60).

### Romidepsin

Romidepsin is a bicyclic depsipeptide HDAC inhibitor (HDACi) administered intravenously at 14 mg/m^2^ on days 1, 8, and 15 of a 28-day cycle, and its AE profile in CTCL has been characterized in two phase II studies (18, 61). Romidepsin does not carry a prominent direct cutaneous toxicity signal in either pivotal trial, which is itself a clinically meaningful observation distinguishing it from other agents such as mogamulizumab or BV. Only 1% of patients in the study by Whittaker et al. experienced dermatitis medicamentosa, though this was a grade 4 reaction (18). Clinicians should maintain systematic skin assessment at each treatment cycle, documenting any changes in the pattern or character of cutaneous findings relative to baseline CTCL involvement.

Gastrointestinal AEs were the prominent drug-related events in both trials. Nausea occurred in the majority of patients and was typically grade 1–2 and responsive to antiemetic prophylaxis with serotonin antagonists (18, 61). Vomiting, diarrhea, anorexia, weight decrease, dysgeusia, and dry mouth were also commonly reported. Fatigue and asthenia were prominent constitutional AEs, peaking in the days following infusion and improving between treatment cycles (18, 61).

Thrombocytopenia and anemia were the most clinically significant hematologic AEs in both studies (18, 61). Neutropenia occurred less frequently than with cytotoxic regimens. Serious adverse reactions including infection, sepsis, and pyrexia were reported in more than 2% of patients, reflecting baseline T-cell immunocompromise in CTCL compounded by myelosuppression. QT/QTc interval prolongation was reported in both studies; in Whittaker et al., QT and corrected QTc prolongation were reported in 3 and 2 patients respectively, with ECG changes returning to baseline within 24 hours and not associated with cardiac arrhythmia (18). Protocol amendments in the Piekarz et al. study mandated electrolyte supplementation to maintain magnesium above 0.85 mmol/L and potassium above 4.0 mmol/L before each dose, recognizing that CTCL patients are at elevated risk for hypomagnesemia and that electrolyte depletion amplifies HDACi-mediated QTc effects (61). Concurrent QT-prolonging medications and CYP3A4 inhibitors should be avoided.

A single CTCL-specific case report reported romidepsin-induced neutrophilic urticaria in a patient with Sézary syndrome, which presented as a reproducible infusion-associated urticarial eruption with near-complete resolution within 6 days (62).

### Vorinostat

Vorinostat (suberoylanilide hydroxamic acid, SAHA) is an oral hydroxamic acid-type HDACi and was the first in its class to be FDA-approved for CTCL. It is given at a standard dose of 400 mg orally once daily.

Alopecia was reported in 17.6% of patients in a pivotal phase IIb trial and 50% of subjects in a long-term tolerability study, reflecting drug effects on rapidly cycling hair follicle keratinocytes (19, 63). In the long-term tolerability analysis, hair growth did not recover in affected patients with alopecia, representing an important finding for patients considering long-term therapy (63). Drug eruption was reported as a cause of treatment discontinuation in the phase II trial (64). In the long-term study, one patient developed a serious cutaneous AE consisting of scaling and scleroderma-like changes of the hands, leading to vorinostat discontinuation and determined by the investigator to be possibly related to the study drug (63).

Gastrointestinal AEs and constitutional AEs were the most commonly reported drug-related events across both pivotal trials. In the phase II trial, fatigue (73%), diarrhea (49%), nausea (49%), dysgeusia (46%), dry mouth (35%), and weight loss (27%) were frequently reported AEs. The phase IIb trial reported diarrhea in 48.6%, fatigue in 45.9%, nausea in 43.2%, anorexia in 25.7%< and dysgeusia in 24.3% of patients, most grade 1–2 (19). Dehydration was the most common serious drug-related AE in the phase II trial, occurring in 11% of patients (64).

Thrombocytopenia was the most clinically significant hematologic AE across studies. In the phase II trial, 54% of patients across regimens experienced thrombocytopenia, with grade ≥3 events in 19% (64). 21.6% of patients experienced thrombocytopenia in the phase IIb trial, and 5.4% of these were grade ≥3 (19). Thromboembolic events were also noted across studies; pulmonary embolism was noted as a grade ≥3 event in 1/6 patients in the long-term tolerability study, and 5.4% of patients had a thromboembolic event in the phase IIb trial. In the phase II trial, 8% of patients had a deep venous thrombosis, while 5% of patients had a pulmonary embolism. Given these events, there should be a low threshold for further evaluation of dyspnea, chest pain, edema, or decreased perfusion in symptomatic patients (19, 63).

### Bexarotene

Bexarotene is a retinoid X receptor-selective retinoid and was the first oral retinoid approved for CTCL based on two pivotal studies: the phase II and III trial in early-stage disease and the multinational phase II-III trial in advanced-stage CTCL, both by Duvic et al. (20, 65) AE in both trials were graded using a descriptive severity scale (Mild/Moderate/Moderately Severe/Severe) rather than the standard NCI CTCAE grading, which limits direct comparison with other agents reviewed here.

Cutaneous AEs were among the most commonly reported drug-related events across both trials. Pruritus was reported in 26% of patients in the early-stage trial and 13% in the advanced-stage trial at 300 mg/m^2^/day, though the relationship between pruritus and drug versus underlying disease activity could not be determined in either study (20, 65). Exfoliative dermatitis was reported in 19% of patients in the early-stage trial and in both dose groups in the advanced-stage trial (20, 65). Rash was reported in 15% of patients in the early-stage trial and 11% at 300 mg/m^2^/day in the advanced-stage trial; “skin disorder” was reported in 14% and 11% respectively (20, 65).

Hypertriglyceridemia was the most frequent and clinically important systemic AE associated with bexarotene, occurring in 79% of all patients in the early-stage trial and 82% at 300 mg/m^2^/day in the advanced-stage trial (20, 65). Lipid elevations occurred rapidly, often within 1–2 weeks of initiating therapy, and were dose-limiting in 43% of all enrolled patients in the advanced-stage trial (65). Extreme triglyceride elevations (>1,300 mg/dL) were associated with acute pancreatitis in 3 patients in the early-stage trial and 1 patient in the advanced-stage trial (20, 65). Hypercholesterolemia accompanied hypertriglyceridemia in both studies.

In the early-stage trial, 41% of patients required levothyroxine supplementation based on symptoms or low free thyroxine levels; monitoring of free thyroxine rather than TSH was recommended for guiding replacement therapy. In the advanced-stage trial, TSH decreased below normal in 54% of patients at 300 mg/m^2^/day within the first 2–4 weeks of therapy. Hypothyroidism and hyperlipidemia both promptly resolved within 1-2 weeks following drug discontinuation.

Leukopenia/neutropenia was dose-related, occurring in 28% of patients in the early-stage trial and constituting 9% of all dose-limiting toxicities in the advanced-stage trial (20, 65). Leukopenia in both studies primarily reflected neutropenia rather than lymphopenia and was not associated with neutropenic fever or sepsis (20, 65). Headache was reported in 47% of patients in the early-stage trial and 20% at 300 mg/m^2^/day in the advanced-stage trial and was among the most commonly reported drug-related AEs (20, 65). Elevations in liver function tests were reported in 10% of patients in the early-stage trial and in 21% of patients at 300 mg/m^2^/day in the advanced-stage trial within the first month of therapy (20, 65).

Case reports highlight metabolic, endocrine, and cutaneous toxicities beyond those captured in pivotal trials. Severe high-density lipoprotein (HDL) depression has been described as a distinct lipid abnormality with cardiovascular implications not captured by triglyceride monitoring alone (66). Reversible pan-hypopituitarism encompassing hypogonadism, hypothyroidism, and hypoadrenalism was reported 5 months after bexarotene initiation in a patient with MF, resolving upon discontinuation and extending the known endocrinologic toxicity profile beyond isolated central hypothyroidism to potentially life-threatening adrenal insufficiency (67). Acute hyperkeratotic and exfoliative erythroderma has been reported and represents a severe paradoxical cutaneous AE requiring differentiation from disease progression (68). Secondary malignancies including extracutaneous lymphoma and Hodgkin lymphoma have also been reported, though causality from bexarotene has not been established (69, 70).

### Denileukin diftitox-cxdl

Denileukin difititox (DD) is an immunotoxin protein composed of amino acid sequences encoding two diphtheria toxin fragments and human interleukin-2 (IL-2) (6). DD binds to cellular IL-2 receptors, leading to it being endocytosed and cleaved to release diphtheria toxins which inhibit protein synthesis and lead to cell death (71). DD was approved in the United States from 1999 to 2014 and was subsequently voluntarily withdrawn from the market to improve its manufacturing. DD-cxdl, the same recombinant protein as DD but reformulated with improved purity and bioactivity, was FDA approved in August 2024.

In a phase III, multicenter trial of patients with relapsed/refractory stage IA-IIIB CTCL treated with DD-cxdl, all but one patient (68/69, 98.6%) had a treatment-emergent adverse event (TEAE), with 43.5% being grade ≥3 and 37.7% requiring inpatient hospitalization or existing hospitalization prolongation (6). TEAEs of special interest, most of which were grade 1–2, included infusion reaction in 73.9%, hypersensitivity in 68.1%, hepatotoxicity in 36.2%, and capillary leak syndrome (CLS)—defined as severely dysregulated fluid homeostasis leading to peripheral edema, hypotension, hemoconcentration, and hypoalbuminemia—in 20.3% (grade ≥3, 5.8%) of patients (6). Notably, there was no evidence of cumulative toxicity, and the incidence of TEAEs, including CLS, decreased with each cycle and remained low with ongoing therapy (6). Other common TEAEs included nausea in 43.5% and fatigue in 31.9% of patients; no deaths were attributed to DD-cxdl in the trial, and only 7.2% of patients discontinued treatment due to TEAEs (6).

To our knowledge, only one death to DD has been reported in the literature, occurring in a patient with cutaneous gamma-delta T-cell lymphoma and undiagnosed cirrhosis who developed fatal capillary leak syndrome and hypoalbuminemia (72).

### Gemcitabine

Gemcitabine is a pyrimidine nucleoside analogue which inhibits DNA synthesis and is generally well tolerated, with primarily hematologic toxicities. Two phase II prospective studies have evaluated the safety and efficacy of gemcitabine in CTCL, the first in 32 treatment-naive patients and the second examining 33 patients with advanced, heavily pre-treated CTCL (7, 8). The most common AE reported were myelosuppression, present in 55% of pre-treated CTCL patients (7). Grade 3 anemia, thrombocytopenia, or leukopenia was present in 13/33 (39%) pre-treated patients, (7) and grade 3 anemia, thrombocytopenia, and neutropenia were observed in 1/32 (3.1%), 4/32 (13%), and 5/32 (16%) of treatment-naive CTCL patients, respectively (8). Among pre-treated patients, one died of febrile neutropenia, and two patients with Sezary syndrome developed hemolytic uremic syndrome (HUS) (7). No patients died of treatment-related complications in the treatment-naive group (8). Beyond hematologic AE, reversible grade 1–3 hepatotoxicity was identified in 12/32 (38%) treatment-naive and 4/33 (12%) pre-treated patients (7, 8).

Reported cutaneous AEs to gemcitabine include flares of lesional erythema in two pre-treated patients within a week of starting gemcitabine, a side effect associated with improved response to treatment, and generalized hyperpigmentation in 6/33 (18%) patients (7). No patients in either phase II study experienced complete alopecia (7, 8).

Real-world, retrospective case reports and series mirror these toxicity profiles with additional characterizations of rare cutaneous AEs. An observational, retrospective study of 24 pretreated, advanced CTCL patients' long-term, fifteen-year outcomes reported no grade ≥3 AEs (73). In a series of 23 patients who received gemcitabine for advanced-stage CTCL, two patients developed severe cutaneous and mucous membrane bullous and erosive dermatitis two days after initiating gemcitabine (74). Other reported rare, gemcitabine-induced cutaneous AEs include one patient who developed linear IgA bullous dermatosis and two reports of Stevens–Johson syndrome/toxic epidermal necrolysis (75–77).

### Pegylated liposomal doxorubicin

Pegylated liposomal doxorubicin (PLD) is a topoisomerase II inhibitor which is liposomally encapsulated to increase circulation and tumor-specific accumulation. PLD is overall well-tolerated in CTCL patients, with primary toxicities including mild myelosuppression, hand-foot syndrome, or palmar-plantar erythrodysesthesia (PPE), and rare, potentially fatal cardiac events.

In a phase II, multicenter, EORTC study of 49 patients with relapsed/refractory advanced MF treated with PLD, grade 4 cardiac events included cardiac ischemia, pulmonary embolism, and allergy/hypersensitivity, each in one patient (9). Other rare grade ≥3 events included constitutional symptoms, gastrointestinal toxicities, and infection, each in 2/49 (4%) patients (9). There were no grade ≥3 hematologic AEs reported (9). The most common grade ≥3 cutaneous toxicity was “hand and foot reaction” in 1 patient (9).

In a second French prospective study of 25 patients with pre-treated stage II-IV MF/SS, the most commonly reported grade 1–2 AE associated with PLD were anemia (32%), nausea/vomiting (20%), and asthenia (16%) (78). Grade 3 AEs included sepsis (12%), and one instance of anemia, asthenia, pneumopathy, and AV block, respectively (78). Cutaneous AEs included PPE in 12% of patients, and one patient developed a grade 3 cutaneous ulceration (78).

A retrospective case series of 24 CTCL patients treated with PLD found that the most common toxicities were low-grade fatigue (41.7%), peripheral edema (25%), and anemia (20.8%) (77). Cutaneous toxicities included hand-foot syndrome and hyperpigmentation, which each affected 4 (16.7%) patients (77). Grade ≥3 toxicities included fatigue in 2 patients and anemia, sepsis, neutropenia, and radiation recall each in 1 patient, respectively (77). Moreover, data supports that PLD is well tolerated as maintenance therapy (79). Of note, the risk of PPE is higher in PLD than conventional doxorubicin due to its long-circulating formulation, enabling the drug to accumulate in the eccrine sweat glands and skin and prolonging its direct toxicity (80).

### Interferon

Interferon alfa and gamma are signaling cytokines with varying immunomodulatory effects which have been used to treat CTCL. However, these have not been evaluated in Phase II studies in the current era of CTCL staging. Peginterferon alfa-2a or ropeginterferon alfa-2b are currently commercially available in the United States for use.

The most common non-cutaneous AEs associated with interferon alfa and gamma in CTCL are flu-like symptoms and elevations in transaminases and lactate dehydrogenase, which tend to be transient, occur early in therapy, and are self-limited (10, 11). Grade 1 hepatotoxicity was reported in up to 60% of patients receiving low-dose IFN-α2b alongside psoralen plus ultraviolet A (PUVA), and grade ≥3 elevations in liver enzymes occurred in 20% of patients (81). Other chronic, dose-related systemic toxicities include anorexia and weight loss, depression, and other neuropsychiatric symptoms (10, 11, 82). Hematologic abnormalities associated with interferon therapy include mild neutropenia, and up to 40% of patients receiving interferon in combination with phototherapy demonstrated leukopenia (10, 81, 82). One patient was reported to develop nephrotic syndrome while receiving interferon gamma (11).

Local injection site reactions are the most commonly reported cutaneous AEs to interferon therapy (10, 11). In addition, one case report detailed a patient with MF who developed discoid-lupus-like lesions at the interferon-alpha injection site, a phenomenon reported otherwise in patients with melanoma and multiple sclerosis receiving interferon (83).

### Alemtuzumab

Alemtuzumab is a humanized immunoglobulin G1 monoclonal antibody targeting CD52, an antigen expressed on malignant B- and T-cells but not hematopoietic stem cells. In the first Phase II study assessing alemtuzumab in 22 patients with advanced stage III-IV MF/SS, the most frequent AEs were infections, hematologic toxicities, and transient infusion reactions, with fever in 68% of patients (12). Serious infectious AEs were reported in 10 (45%) patients: four (18%) developed cytomegalovirus (CMV) reactivation after presenting with fever and subsequently responded to ganciclovir treatment, three patients developed fevers of unknown origin, and generalized herpes simplex virus, fatal aspergillosis, and fatal Mycobacterium pneumonia were each reported in one patient, respectively (12). Notably, all serious infectious AEs except CMV reactivation occurred in patients who had received at least 3 prior regimens, underscoring the risk of compounding immunosuppression in heavily pretreated patients (12). All patients developed anemia, 95% of which were grade 1, neutropenia, of which four (18%) were grade 4 reactions, and thrombocytopenia, of which 82% were grade 1 (12). Transfusion reactions were common, mostly mild to moderate in severity, and for which over half of patients (59%) received intravenous corticosteroids as secondary prophylaxis during the first week of treatment (12).

Supportive of the previous Phase II study's findings, a prospective study of 14 patients receiving low-dose subcutaneous alemtuzumab showed a statistically significant association between higher doses and infection risk: infectious complications occurred in all four patients treated with higher dose alemtuzumab (up to 15 mg) and no patients treated with a maximum dose of 10 mg (84).

Several rare complications of alemtuzumab have been described in case reports and series. A novel hypersensitivity reaction was described in a series of five patients receiving low-dose subcutaneous alemtuzumab, presenting as persistent pruritic erythematous papules and plaques with evidence of subacute spongiotic dermatitis and endothelial activation on histology, potentially mimicking recurrent CTCL (85). In addition, several unusual infections have been reported, including severe parvovirus infection, JC virus encephalitis, and EBV-positive lymphoproliferative disease (86–88).

Secondary malignancies and large cell transformation have also been reported in patients receiving alemtuzumab for CTCL. In the aforementioned Phase II study, one patient developed progression of pre-existing squamous cell skin carcinoma during treatment (12). In the largest retrospective series of 39 patients with advanced MF/SS, five patients experienced cutaneous large T-cell transformation during alemtuzumab treatment, and 1 patient developed a secondary primary cutaneous large B-cell lymphoma (89). In a series of 19 heavily pre-treated patients with erythrodermic CTCL, one patient developed concurrent mantle cell lymphoma 6 months after completing alemtuzumab therapy (90). Lastly, a case report described transformation of Sézary syndrome into CD30 + anaplastic large T-cell lymphoma (ALCL) 3 weeks after initiating alemtuzumab, with matching T-cell clones confirmed across the SS, ALCL, and MF lesions (91).

### Pembrolizumab

Pembrolizumab is an immunoglobulin G4, anti-PD-1 monoclonal antibody that acts as an immune checkpoint inhibitor (ICI) to induce robust anti-tumor responses across a variety of malignancies. PD-1 expression has been demonstrated in MF and to a lesser extent SS, prompting consideration of using ICIs to treat CTCL (92).

The CITN-10 Phase II trial evaluated pembrolizumab in 24 patients with relapsed/refractory MF and SS (13). Notably, four (17%) patients discontinued pembrolizumab due to development of grade ≥3 immune-related AEs, including pneumonitis, duodenitis, transaminitis, and corneal ulcers (13). Over half (53%) of patients with SS experienced cutaneous flare reactions, defined as worsened erythema and pruritus, which were self-limiting and did not result in treatment discontinuation (13). These flares correlated with high PD-1 expression on Sezary cells but did not predict subsequent clinical response to pembrolizumab, unlike in melanoma (13).

In a real-world retrospective series of 30 patients, immune-related AEs were observed in 43% of patients, which were most commonly cutaneous, endocrine, and gastrointestinal and grade 1–2, with no grade ≥4 events (93). Risk of disease progression is of particular concern in T-cell malignancies; the NCCN guidelines caution that rapid progression has been reported in Human T-cell Lymphotropic Virus (HTLV)-positive CTCL patients receiving pembrolizumab (3, 94). Although uncommon in CTCL relative to other T-cell lymphoma subtypes, a case report demonstrated that anti-PD-1 therapy activated CD4 + malignant T-cells harboring somatic PRKCQ amplification and subsequently lead to disease hyperprogression (94). Mechanistically, PD-1 blockade has been proposed to paradoxically accelerate tumor proliferation by releasing the tumor-suppressive function of PD-1 on malignant T-cells (94). Therefore, clinicians must distinguish transient, immunotherapy-related cutaneous flares from true disease progression for which PD-1 therapy should be discontinued.

### Temozolomide

Temozolomide is an oral alkylating agent which induces cytotoxic DNA damage primarily through methylation. Low expression of the DNA repair protein MGMT in malignant CD4^+^ T-cells in MF/SS may increase sensitivity to temozolomide, supporting its use in CTCL (14). Across two phase II trials in which a combined 35 patients with pretreated MF/SS received temozolomide, the majority of AEs were grade 1–2 and transient (14, 95). Pain/pruritus and fatigue were the most common side effects reported in the larger, multicenter trial, occurring in 20/26 (76.9%) and 15/26 (57.7%) patients, respectively (14). Hematologic toxicity was the primary dose-limiting toxicity: in the larger trial, grade 1–3 thrombocytopenia was the most frequent hematologic event, occurring in 10 (38.5%) patients, followed by grade 2–3 neutropenia in four (15.3%) patients, and grade 2 lymphopenia in 2 (7.7%) patients (14). In the smaller phase II trial of nine patients, grade 3 thrombocytopenia and grade 3 neutropenia each caused a one-week treatment delay in one patient (95).

Cutaneous AEs included low grade rash or erythema and alopecia, with one grade 3 skin reaction leading to treatment discontinuation across phase II studies (14, 95). Treatment was discontinued due to toxicity in 3/26 patients (12%) in the larger trial for grade 3 thrombocytopenia, lymphopenia, and skin reaction, respectively, whereas all nine patients in the smaller trial completed the planned three courses of temozolomide (14, 95). No treatment-related deaths were reported in either study, and no rare idiosyncratic reactions described in the broader temozolomide literature have been reported in the CTCL population to date.

## Discussion

Systemic therapies for MF and SS possess distinct AE profiles that should inform patient counseling, treatment selection, monitoring, and supportive care. Table 2 compares the characteristic toxicities, mimickers of CTCL progression, timing of toxicities, and practical management considerations which vary substantially by drug class. Understanding the characteristic toxicities and their timing, such as BV's defining limitation of peripheral neuropathy, mogamulizumab's characteristic rash and MAM/Mc, pralatrexate's dose-dependent stomatitis/mucositis, hypertriglyceridemia and central hypothyroidism that may arise early in bexarotene treatment, and hepatoxicity associated with interferon or methotrexate, may aid providers in navigating the nuances of systemic treatment selection and counseling for CTCL patients.

**Table 2:** Comparison of key AEs and patient management considerations across established MF/SS systemic therapies.

Agent	Characteristic AE(s)	Mimics CTCL progression?	Timing of toxicities	Practical considerations for management
Brentuximab vedotin	Peripheral neuropathy, associated with the highest reported toxicity-related discontinuation rate (19%)	Rare; cutaneous AEs are generally mild	Late/cumulative MMAE-related cutaneous toxicities have been reported	Peripheral neuropathy is the key dose limiting toxicity
Mogamulizumab	Mogamulizumab-associated rash (MAR); myocarditis/myositis (MAM/Mc)	Yes; MAR can closely mimic CTCL	Treatment-emergent MAR may recur on rechallenge; MAM/Mc is typically delayed and may onset even after mogamulizumab discontinuation	MAR requires careful clinicopathologic examination to distinguish from CTCL progression; monitor for MAM/Mc
Pralatrexate	Stomatitis/mucositis	No	Stomatitis/mucositis is dose-dependent	Supplement with folic acid; assess mucositis before each dose and hold treatment for grade ≥2 stomatitis/mucositis; 15 mg/m^2^ dosing improves tolerability
Methotrexate	Hepatotoxicity; mucocutaneous ulceration; myelosuppression	Rare; ulceration or skin necrosis may mimic CTCL lesions	Toxicities are typically delayed and cumulative	Supplement with folic acid; monitor for hepatic, renal, hematologic, and mucocutaneous toxicities
Romidepsin	Gastrointestinal and constitutional symptoms; QT/QTc abnormalities	No	Fatigue/asthenia peaks after infusion and improves between cycles; ECG changes reported to be reversible within 24 h	Check and correct for electrolyte abnormalities; avoid QT-prolonging drugs and CYP3A4 inhibitors
Vorinostat	Gastrointestinal symptoms; thromboembolism; alopecia	No	No recovery of alopecia long-term	Counsel on chronic alopecia; maintain low threshold to work-up thromboembolic symptoms
Bexarotene	Hypertriglyceridemia, central hypothyroidism	Yes; exfoliative rash or erythroderma may mimic CTCL progression	Toxicities arise early—hyperlipidemia within 1–2 weeks, thyroid abnormalities within 2–4 weeks—and are reversible upon drug cessation	Monitor and provide prophylaxes (levothyroxine, statins) against endocrinologic and metabolic toxicities
DD-cxcl	Infusion or hypersensitivity reactions; capillary leak syndrome (CLS); hepatotoxicity	No	AEs decrease in frequency with each cycle with no cumulative toxicity reported	Monitor for edema, hypoalbuminemia, hypotension, and hemoconcentration, which may indicate CLS
Gemcitabine	Myelosuppression; reversible hepatotoxicity	Rare; early lesional erythema flares may mimic CTCL progression	Transient, lesional flares typically arise within 1 week of drug initiation	Generally well-tolerated; heavily pre-treated patients may be less able to tolerate myelosuppression
Pegylated liposomal doxorubicin	Hand-foot syndrome/palmar-plantar erythrodysesthesia (HFS/PPE); mild myelosuppression; rare, serious cardiac events	Yes; toxic erythema of chemotherapy, including HFS/PPE, must be distinguished from CTCL progression	Cumulative direct skin toxicity increases with duration of exposure	Generally well-tolerated; monitor for and treat HFS/PPE
Interferon	Highest reported rates of hepatotoxicity; flu-like symptoms; chronic neuropsychiatric symptoms	No	Early transient and self-limited flu-like symptoms; chronic, dose-related anorexia, weight loss, and depression	Use caution in elderly patients due to risk of weight loss and anorexia worsening frailty; consider risk of neuropsychiatric side effects
Alemtuzumab	Serious/opportunistic infections; hematologic toxicity	Yes; hypersensitivity rashes may mimic CTCL	Infusions reactions tend to be transient; infection risk is dose-dependent and increases with compounded prior immunosuppression	Take caution in heavily pre-treated or immunosuppressed patients due to infection risk
Pembrolizumab	Immune-related AEs (irAEs) led to treatment discontinuation in 17% of patients; transient cutaneous flares	Yes; cutaneous immune-related AEs must be distinguished from CTCL progression	Cutaneous flares are transient and self-limiting; irAEs can occur at any time after initiation	Cutaneous irAEs require assessment to distinguish between CTCL flare versus de-novo rash
Temozolomide	Hematologic toxicity; pain/pruritus; fatigue	No	Mostly low-grade and transient toxicities	Marrow toxicity may be dose-limiting

A recurring challenge in MF/SS is that many cutaneous AEs may resemble active or progressive disease, particularly for patients undergoing treatment with mogamulizumab, bexarotene, PLD, alemtuzumab, and pembrolizumab. This diagnostic dilemma may lead clinicians to misattribute drug toxicity to CTCL progression, prompting unnecessary discontinuation of a potentially effective therapy, whereas misclassifying CTCL progression to drug toxicity may thwart or delay initiation of appropriate treatment. Careful baseline documentation of patients' skin findings, longitudinal clinical assessment, clinicopathologic correlation, and an understanding of therapy-specific toxicity patterns are therefore essential to optimizing care for these patients.

As there is currently no cure for CTCL, each patient's treatment should be individualized based on their overall goals of therapy and toxicity profile (3). The median age at CTCL diagnosis is in the fifth to sixth decade (96), and thus it is important to consider which agents are best suited for patients who are frail, elderly, or already heavily immunosuppressed. Agents with potential for severe myelosuppression, such as gemcitabine, or with high reported rates of infectious AE, such as alemtuzumab, should be used cautiously in at-risk populations. Additionally, interferon's associated systemic symptoms, including anorexia, may worsen frailty in elderly patients; providers may also consider screening patients for psychiatric or neurologic comorbidities such as depression or dementia due to interferon's associations with neuropsychiatric AEs. While methotrexate should be avoided in patients with existing hepatic disease or alcohol use disorder, its side effect profile may be overall gentler and less immunosuppressive in an elderly or frail cohort without such comorbidities. Lastly, while this review is limited to AEs associated with systemic agents, skin-directed therapies are important first-line treatments and may be more appropriate for patients who are vulnerable or unable to tolerate systemic toxicities.

Future studies may seek to standardize AE reporting in MF/SS, better characterize the nuanced morphologies and patterns of cutaneous toxicities, and generate vital patient-centered outcome measures such as pruritus, quality of life, functional impairment, and treatment adherence. A comprehensive understanding of both common and rare toxicities can help clinicians individualize systemic therapy, anticipate complications, and preserve quality of life for patients receiving long-term treatment for MF and SS.
